# Translating From Egg- to Antigen-Based Indicators for *Schistosoma mansoni* Elimination Targets: A Bayesian Latent Class Analysis Study

**DOI:** 10.3389/fitd.2022.825721

**Published:** 2022-02-18

**Authors:** Jessica Clark, Arinaitwe Moses, Andrina Nankasi, Christina L. Faust, Moses Adriko, Diana Ajambo, Fred Besigye, Arron Atuhaire, Aidah Wamboko, Candia Rowel, Lauren V. Carruthers, Rachel Francoeur, Edridah M. Tukahebwa, Poppy H. L. Lamberton, Joaquin M. Prada

**Affiliations:** 1 Wellcome Centre for Integrative Parasitology, Institute of Biodiversity, Animal Health & Comparative Medicine, University of Glasgow, Glasgow, United Kingdom; 2 Vector Control Division, Ministry of Health, Kampala, Uganda; 3 Faculty of Science and Engineering, University of Chester, Chester, United Kingdom; 4 Faculty of Health and Medical Sciences, University of Surrey, Guildford, United Kingdom

**Keywords:** diagnostics, EPHP, POC-CCA, intestinal schistosomiasis, NTDs, neglected tropical diseases, gold standard, G-Score

## Abstract

Schistosomiasis is a parasitic disease affecting over 240-million people. World Health Organization (WHO) targets for Schistosoma mansoni elimination are based on Kato-Katz egg counts, without translation to the widely used, urine-based, point-of-care circulating cathodic antigen diagnostic (POC-CCA). We aimed to standardize POC-CCA score interpretation and translate them to Kato-Katz-based standards, broadening diagnostic utility in progress towards elimination. A Bayesian latent-class model was fit to data from 210 school-aged-children over four timepoints pre- to six-months-post-treatment. We used 1) Kato-Katz and established POC-CCA scoring (Negative, Trace, +, ++ and +++), and 2) Kato-Katz and G-Scores (a new, alternative POC-CCA scoring (G1 to G10)). We established the functional relationship between Kato-Katz counts and POC-CCA scores, and the score-associated probability of true infection. This was combined with measures of sensitivity, specificity, and the area under the curve to determine the optimal POC-CCA scoring system and positivity threshold. A simulation parametrized with model estimates established antigen-based elimination targets. True infection was associated with POC-CCA scores of ≥ + or ≥G3. POC-CCA scores cannot predict Kato-Katz counts because low infection intensities saturate the POC-CCA cassettes. Post-treatment POC-CCA sensitivity/specificity fluctuations indicate a changing relationship between egg excretion and antigen levels (living worms). Elimination targets can be identified by the POC-CCA score distribution in a population. A population with ≤2% ++/+++, or ≤0.5% G7 and above, indicates achieving current WHO Kato-Katz-based elimination targets. Population-level POC-CCA scores can be used to access WHO elimination targets prior to treatment. Caution should be exercised on an individual level and following treatment, as POC-CCAs lack resolution to discern between WHO Kato-Katz-based moderate- and high-intensity-infection categories, with limited use in certain settings and evaluations.

## Introduction

Schistosomiasis, caused by a parasitic helminth, is endemic in 54 countries, infecting over 240 million people and has the second greatest socio-economic impact of any parasitic disease after malaria (1) with several million people experiencing severe morbidity despite nearly two decades of interventions (2). Around 90% of cases are found on the African continent, caused by *Schistosoma mansoni* and *Schistosoma haematobium*, causing intestinal and urogenital schistosomiasis respectively.

The World Health Organization (WHO) has targeted schistosomiasis for elimination as a public health problem (EPHP) by 2030 (3). This goal is achieved when there are <1% “heavy-intensity” infections in a target population. For *S. mansoni*, “heavy” is ≥400 eggs per gram of stool (epg), when measured by Kato-Katz from stool samples (4). However, these thresholds are problematic because they assume that the egg count is linearly related to the unobservable infection intensity (adult worm density) and morbidity, and because Kato-Katz lack sensitivity and show significant within- and between-sample and -day variation (5, 6). This categorization is therefore unlikely to be static in time, and underappreciates the contribution of light or moderate intensity infections to morbidity and transmission. These guidelines are also in contrast to community risk categories, which are given in terms of infection prevalence, not intensity (7).

In 2017, the WHO endorsed the urine-based point-of-care circulating cathodic antigen diagnostic (POC-CCA) (7), which detects *S. mansoni* antigens (8). The POC-CCA is more sensitive and can detect infections missed by the Kato-Katz (juveniles and non-reproducing worms), but may also lack specificity (9, 10). Current WHO guidance suggests a 10% difference between egg- and antigen-based prevalence estimates in high prevalence settings (7), but a 20% discrepancy in low- and moderate-prevalence settings. The evidence supporting this is unclear, with recent work in a high-risk community also suggesting a 20-30% difference (11). There is also no indication of how POC-CCA aligns with infection intensity-based targets, which is desperately needed to efficiently harness POC-CCA’s higher sensitivity (12).

The POC-CCA has traditionally been scored as Negative, Trace, +, ++ or +++ (referred to as POC-CCA+ from here on) as a semi-quantitative presumption of infection intensity, based on the colored response on the lateral flow assay. However, there is ongoing debate regarding the interpretation of Trace scores, as positive or negative, which leads to divergent epidemiological and drug-efficacy estimates (13, 14), and hinders analysis because strong interpretation assumptions must be made (15–17). To overcome this dilemma, the G-Score method was recently developed (18). Diagnostic cassettes are compared to 10 dummy cassettes, pre-labelled with reference scores from G1 (negative) to G10 (highest positive score), with the aim of reducing inter-reader differences and increasing resolution across the scores (18, 19). A score of G2 or G3 is supposedly equivalent to Trace, however, with no schistosomiasis diagnostic gold standard, it is still unclear whether Trace, and therefore G2 and G3, are negative or positive, and there is no indication of how the G-Scores relate to true infection intensities. It is also hard to ascertain how the G-Score performs in terms of sensitivity or specificity in comparison to the POC-CCA+.

The overall aim of this study was to improve the interpretation and utility of the POC-CCA, to help guide policy, enabling countries to make informed decisions for *S. mansoni* control. We did this by: 1) Determining how infection intensity relates to the POC-CCA scores; 2) Estimating the probability of infection, particularly those associated with Trace and G2 or G3 scores; 3) Quantifying and comparing the performance of G-Score and POC-CCA+ methods; and 4) Assessing the expected distribution of POC-CCA scores in EPHP settings, through a simulation study, to determine an analogous POC-CCA threshold to Kato-Katz egg counts.

## Methods

### Study Design, Enrolment and Participants

The data used in this modelling study were collected pre-praziquantel treatment, and three-weeks, nine-weeks and six-months post-treatment, from September-March 2017/18. 220 randomly selected children of equal sex distribution aged 6-14 were enrolled into the study from Bugoto Lake View Primary School, Mayuge District, Uganda. Ten students provided no samples leaving a cohort of 210. A full description of the demographic breakdown of the cohort and estimated infection prevalence is provided elsewhere (11). Children present at each timepoint provided stool samples on three consecutive days with duplicate Kato-Katz smears made per stool, giving up to six Kato-Katz smears per timepoint/child. A single POC-CCA test was performed on one urine sample per timepoint/child, scored with the POC-CCA+ and G-Score methods. Observed treatment with praziquantel was administered at 40mg/kg alongside a carbohydrate-based meal (20).

### Ethical Clearance

Ethical Approval was granted from the Vector Control Division Research Ethics Committee (VCDREC/062), Uganda National Council of Science and Technology (UNCST-HS 2193) and University of Glasgow Medical, Veterinary and Life Sciences Research Ethics Committee (200160068). Informed consent was given by signature or thumb print, prior to data and sample collection, by the parent/legal guardian of all recruited children and informed assent from all children aged eight and older.

### Model Structure

We adapted an existing latent class model framework which considers the Kato-Katz and POC-CCA data for each individual as imperfect estimators of an individual’s latent infection status (11). Specific details including the handling of missing data are in the supplementary material. Two models are presented: one with Kato-Katz and POC-CCA+ and one with Kato-Katz and G-Score. We used raw repeated Kato-Katz counts (eggs counted on each slide) and POC-CCA scores, transformed such that the POC-CCA+ and G-Scores were from 0-4 and 0-9 respectively. We assumed the POC-CCA results were related to infection intensity through a logistic function in the likelihood function for the POC-CCA data. The real number of the denominator was the true latent infection intensity as estimated by the model, and the numerator, the highest integer value of the POC-CCA scoring method. This meant that heavily infected individuals could have higher POC-CCA test results, but with no strong assumptions on the interpretation of Trace or G2/G3 scores as has previously been necessary (15, 17, 21, 22). Analysis and visualizations were produced using R version 4.0.2 (23) and models fit with the *runjags* (24) package.

#### Relating POC-CCA Scores to True Infection Intensity

We reconstructed the form of the logistic function to visualize the relationship between true infection intensity and expected POC-CCA scores for POC-CCA+ and G-Score. This was performed by randomly sampling from model parameter posterior distributions for each POC-CCA scoring method.

#### Interpretation of Trace and G2/G3

Each iteration of the model runs, allocates an infection status to each individual. By averaging over the total number of iterations for each model, we determined for each individual the time-specific probability of being infected. Using the individuals with 0 epg when measured by Kato-Katz, we correlated each individual’s estimated probability of true infection with their POC-CCA score.

#### Assessing the Performance of POC-CCA+ Versus G-Score

Calculated using the individual-level true infection status, as estimated by the model, we produced Receiver Operator Characteristic (ROC) curves (25) to compare the performance of the POC-CCA+ and G-Score methods at each timepoint. The overall performance of the two diagnostics were quantified with the Area Under the Curve (AUC) value, which, in this instance, is equal to the Wilcoxon-Mann-Whitney test statistic and describes the relationship between the true-positive (sensitivity) and false-positive (1-specificity) rates. We compared the ROC curves with the probability of infection to infer an optimal threshold for disease diagnosis.

#### Estimating the EPHP Target for Antigen-Based Diagnostics

We conducted a simulation study to determine a target for the POC-CCAs, analogous to the current Kato-Katz-based WHO EPHP target. Using 100 prevalence values ranging from 1-10% (in line with WHO low community-risk categorization (7) based on Kato-Katz counts, and where EPHP is most likely to be first reached), we simulated POC-CCA+ scores and G-Scores for each prevalence level, simulating 50 target populations with 10,000 individuals each. Each individual was assigned an infection status (infected or not infected). The allocation of POC-CCA scores replicated the models. Details of the simulation can be found in the supplementary material file.

## Results

**Figure 1:**
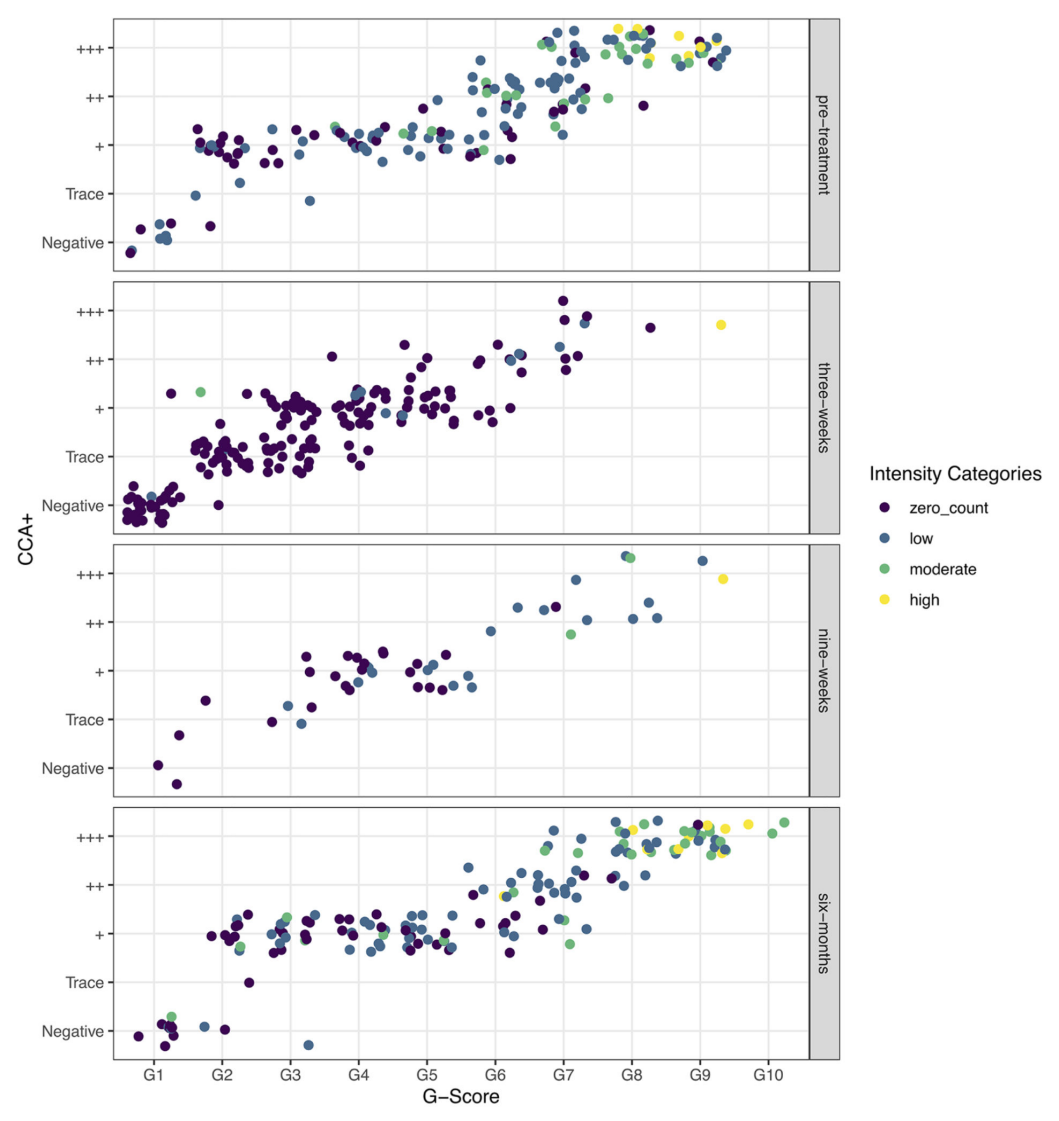
Raw data showing the correlation between G-Scores and POC-CCA+ colored by the WHO *Schistosoma mansoni* infection-intensity categories, as determined by Kato-Katz egg counts, and split by the timesteps used in the models: pre-treatment, and three-weeks, nine-weeks and six-months post-treatment.

Visualizing the raw data used to parametrize the models, it is evident that at all timepoints there is a positive association, as previously shown (18), between the POC-CCA+ and G-Score scoring method (Figure 1). However, whilst heavy intensity infections aggregate largely between G7-G10 or +++, zero epg by Kato-Katz and low intensity infections (1–99 epg) are distributed across all POC-CCA+ and G-Scores, at all timepoints, such that in the field there would be no indication of infection intensity from just the POC-CCA scores.

**Figure 2:**
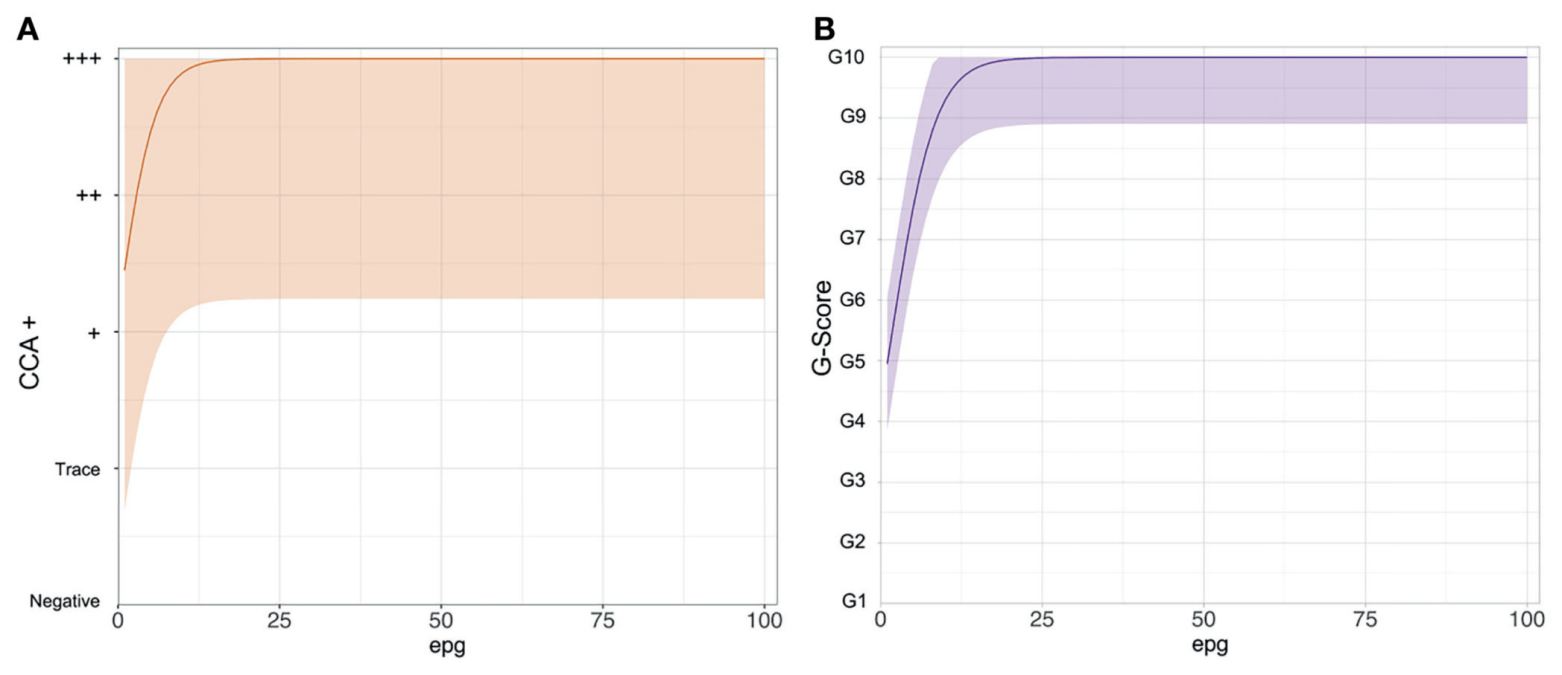
The functional form of the logistic curves showing the relationship between the point-of-care circulating cathodic antigen score and true *Schistosoma mansoni* infection intensity as eggs per gram of stool (epg). **(A)** POC-CCA+, **(B)** G-Scores.

This is because the POC-CCAs saturate at very low true infection intensities (Figure 2). The logistic curve shows that for those who are definitely infected, true infection intensities (intensities not necessarily captured by Kato-Katz) as low as 1 epg, could illicit a score between Trace and +++ (Figure 2A), and between G4 and G6 (Figure 2B). The G-Score reaches its maximum score of G10 by, on average, a true infection intensity of 25 epg.

**Figure 3:**
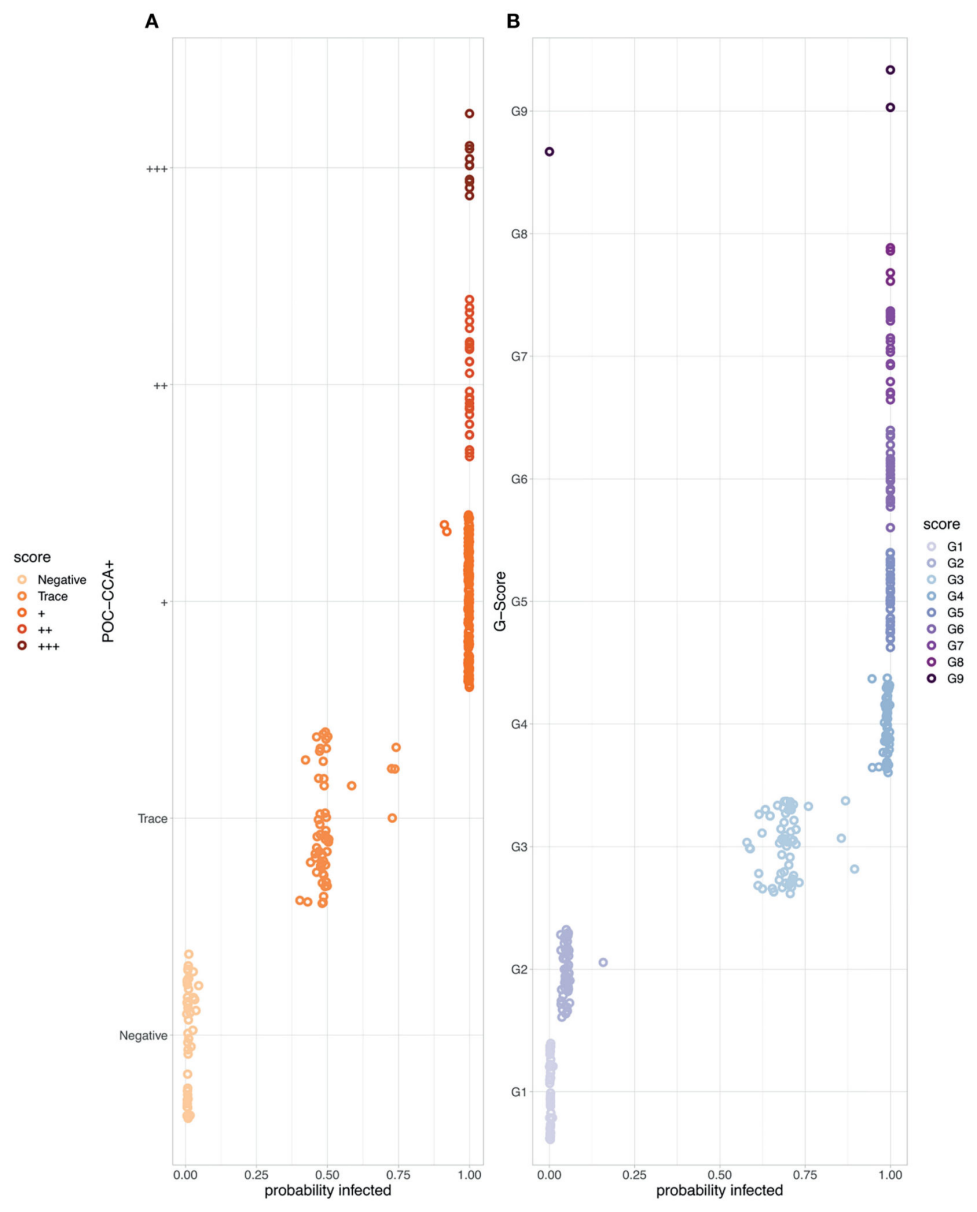
The probability of *Schistosoma mansoni* infection associated with each point-of-care circulating cathodic antigen score where the corresponding egg count was zero. **(A)** POC-CCA+ **(B)** G-Scores. Note that there were no scores of G10 given to those with zero egg counts.

We show the probability of infection associated with each POC-CCA score (Figure 3). For the POC-CCA+, the percentage probability of infection for a Trace score aggregates around 50%, whilst + and above is indicative of almost certain infection. This aggregation around 50% for a Trace score indicates maximum uncertainty in the score allocation. For the G-Score method, a score of G2 has only a very low probability of infection associated with it, whilst G3 indicates a 62-75% probability of infection.

**Figure 4:**
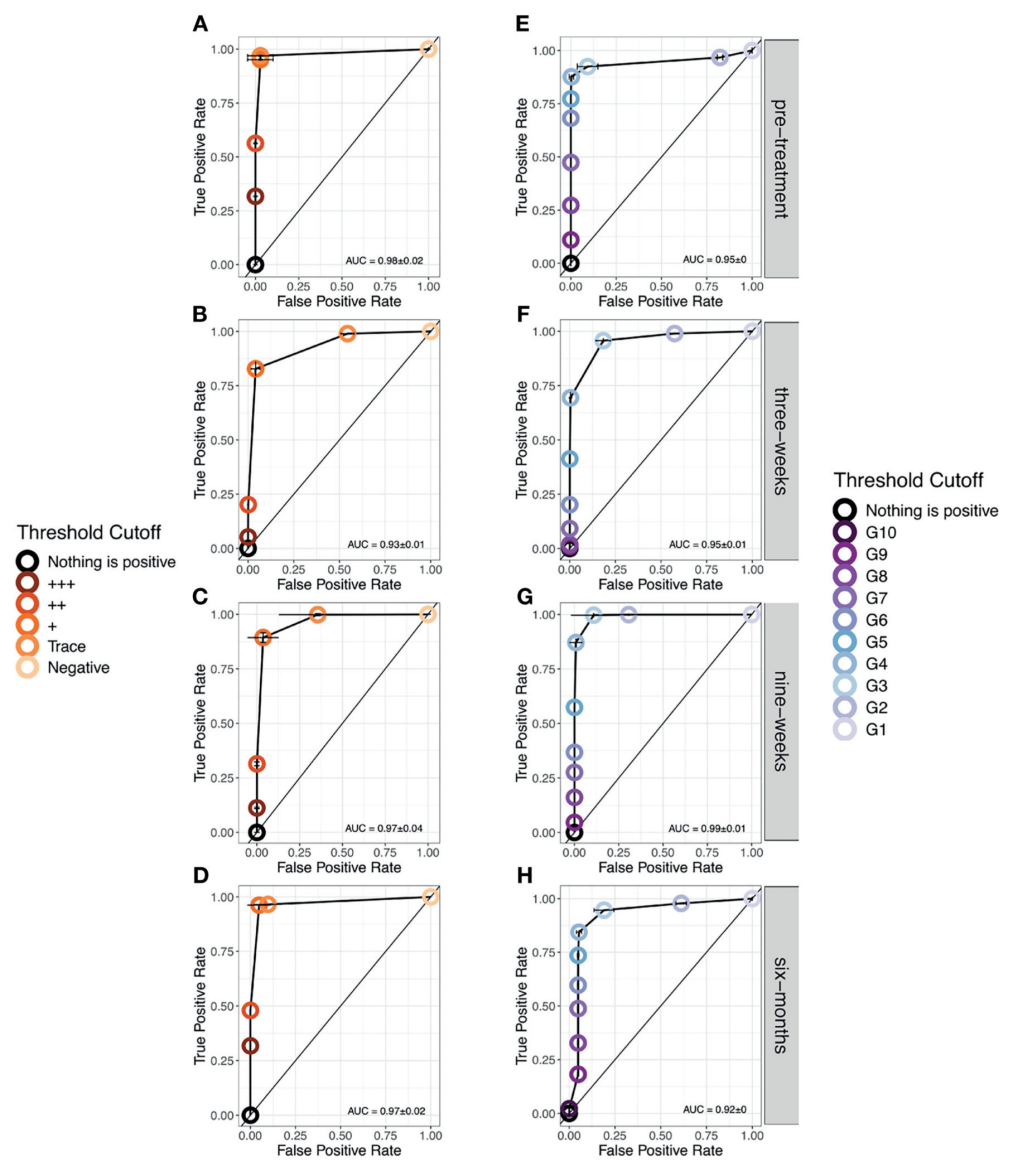
Receiver Operator Characteristic (ROC) curves showing the performance of each diagnostic scoring method at each timestep. **(A–D)**. POC-CCA+ **(E–H)**. G-Scores. Note that only at six-months post-treatment, was a score of G10 given.

As there were no Trace scores allocated pre-treatment and six-months post-treatment, there is no indication for whether this threshold would impact the AUC at these timepoints. However, at three- and nine-weeks post-treatment using Trace as the earliest positive diagnosis maintains sensitivity but with a false positive rate around 50% (Figures 4A–D) reflecting the uncertainty in the probability of infection. The use of + as the threshold reduces sensitivity by around 20% but this reduces the high false positive rate to almost 0 across all timepoints. Regarding G-Scores, taking a G2 score to be positive would provide a highly variable false positive rate (30-80%) in relation to time since treatment. The severity of this false positive rate reduces with treatment but is seen again by six-months post-treatment. A score of G3 and above as the cut-off point provides a lower, more stable false positive rate, ~10-18%, whilst maintaining a high sensitivity (~90%), again depending on time since treatment (Figures 4E–H).

**Figure 5:**
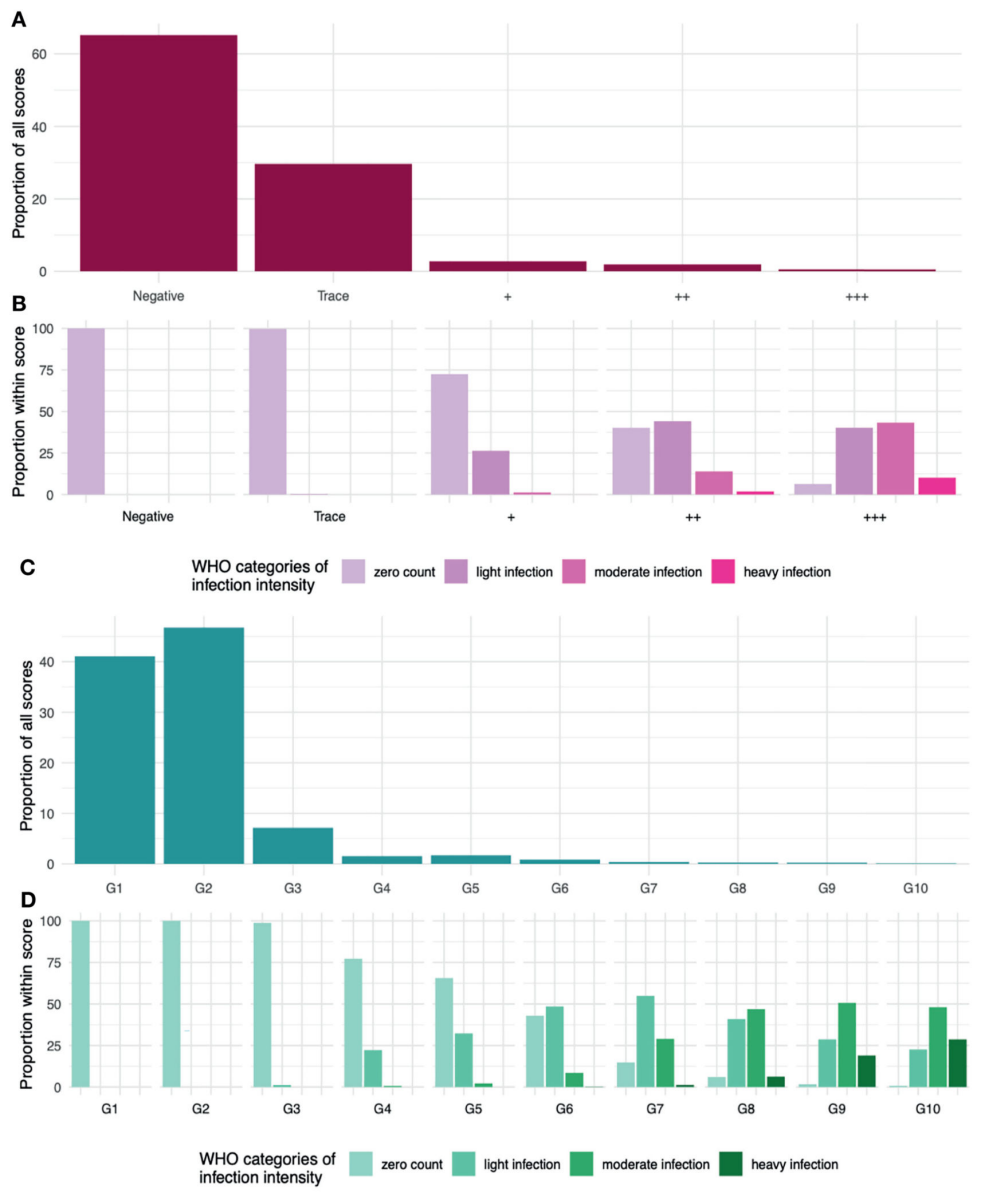
**(A)** The distribution of given POC-CCA+ scores from a simulation of 100,000 people in 50 infected populations. **(B)** the distribution of WHO infection-intensity categories across the given scores. Zero counts in light purple. Light/Low infection intensities in dark purple. Moderate in fuchsia pink and heavy infection intensity in neon pink. **(C)** The distribution of given G-Scores scores. **(D)** the distribution of WHO infection-intensity categories across the given scores. Zero counts in sea foam green. Light/Low infection intensities in light green. Moderate in tree green and heavy infection intensity in dark green.

Though Kato-Katz epg and POC-CCA scores cannot be directly aligned, we can observe the distribution of WHO infection-intensity categories based on Kato-Katz egg counts across the POC-CCA scores (Figures 5A, C). Heavy intensity infections are found in the POC-CCA categories ++ and +++. These categories amount to 2·3% of the total POC-CCA+ scores (Figure 5A). We therefore propose this as a threshold, such that if a target population has ≤ 2·3% ++ or +++, it is likely EPHP has been achieved. Similarly for G-Score (Figures 5B, D), heavy intensity infections are found from G7 upwards, of which these categories make up 0·92% of the allocated scores. We therefore propose that if a target population has ≤0·92% G7 and above, it will likely have achieved EPHP.

## Discussion

We present a quantitative analysis of *S. mansoni* diagnostic data to improve interpretations of the more sensitive, but not 100% specific, POC-CCA. We show, for the first time, that POC-CCA+ and G-Scores are not associated with a particular intensity of *S. mansoni* infection, because the POC-CCA test itself saturates at low infection intensities. We also show that in a high prevalence setting, the probability of infection cannot be estimated robustly for those with Trace scores. However, nearly three quarters of those with a G3 score are likely to be infected and that it is highly probable that a score of G2 reflects a true negative diagnosis. Using ROC curves, we show that considering Trace as positive will result in a high proportion of false positives, whilst G3 would produce far fewer false positives with little reduction in sensitivity. Most importantly, we show that if a population has ≤2·3% of POC-CCA+ scores of ++ and +++, or ≤0·92% of G7 and above then it is highly likely that the target population has achieved WHO’s EPHP definition of <1% heavy infections.

Our results provide quantitative evidence that at baseline (pre-treatment) the G2-G3 boundary provides a sensitive and specific cut off and we therefore recommend that G3 and above be considered positive. However, post-treatment this relationship changes. There is an alteration in clinical sensitivity and specificity that suggests a change in the biological relationship between the production and/or excretion of the eggs and antigens (captured in the model as a change in the shape of the logistic curve), rather than a change in the technological sensitivity and specificity. This is most likely due to the number of eggs being excreted per adult worm present, changing with treatment. This could also be due to juvenile worms surviving treatment and continuing to regurgitate antigens but not yet producing eggs, or previously egg-producing adult worms surviving treatment but becoming – at the very least, temporarily – sterilized (26) or otherwise unable to reproduce (27).

Having recognized this, we attempted to estimate by time, the *k* and *intercept* parameters that form the shape of the logistic function in the POC-CCA likelihoods. However, there were insufficient data to do this at individual timepoints. In future studies, larger sample sizes will be needed to sufficiently power the study to be able to estimate the shape of this relationship as a function of time post-treatment. *Post-hoc* investigative analyses showed that there were no significant differences between pre-treatment and at six months post-treatment, indicating that any biological perturbation in this relationship, caused by praziquantel treatment, has returned to that of pre-treatment by six-months post-treatment.

Both of our findings are novel, and due to their importance for diagnostic interpretation should be reflected in WHO guidelines: Pre-treatment, G3 and above should be considered positive. However, post-recent-treatment, POC-CCA scores cannot be compared against historical drug efficacy measures of egg reduction rates, not because the POC-CCA lacks accuracy post treatment, but because the POC-CCA scores do not correlate to egg excretion in the same biological way and therefore cannot be used as a proxy of infection intensity reduction in the same way. However, they may be better measures of drug efficacy, but not as it has been historically viewed. Additionally, this work may indicate that Kato-Katz cannot accurately measure drug efficacy on the adult worms, and therefore are not the measures we should be guided by. The greater sensitivity gained by using POC-CCA tests may however enable a better understanding of who is infected and what proportion of people are contributing to transmission. However, it should be noted that it is the eggs that are excreted that contribute to transmission and therefore they will always still be of importance to accurately quantify. Conversely it is the eggs that are not excreted (i.e., those that cannot be detected by Kato-Katz) that contribute to morbidity, which is what the WHO aims to reduce, further highlighting the complexity of accurately diagnosing infections and morbidity without a gold standard for either.

Recent evidence has shown that the WHO Kato-Katz-based infection-intensity categories do not correlate to morbidity, with low and moderate intensity infections also causing significant morbidity (28). This suggests that EPHP measured by Kato-Katz will not be enough to truly reduce the observed levels of morbidity. Low and moderate intensity infections are found in the ++ and +++ POC-CCA+ categories, and in G7 and above, in our simulated EPHP target populations. We therefore propose more conservative, and logistically easier, cut offs of ≤2% of ++ and +++, or ≤0·5% of G7 and above, which means our proposed indicators of EPHP when using either POC-CCA scoring method, may reduce the prevalence and severity of morbidity further than the egg-based metric. However, more must be done to understand how morbidity manifests for those with low intensity infections, or those that have infections undetectable by Kato-Katz but with low POC-CCA scores. For example, it is common for adults to exhibit greater morbidity, but lower egg counts, than children, likely from long-lasting, untreated chronic infections (29). Models have recently shown that in some settings, reaching these morbidity targets and reactively reducing treatment frequency could result in recrudescence (30). suggesting the morbidity targets are not an optimal stepping stone and that the reduction of prevalence as suggested elsewhere (31) will be more effective.

To conclude, we present, for the first time, policy recommendations for the use of the antigen-based POC-CCA diagnostic to identify WHO 2030 EPHP targets. We advocate for the use of the newer G-Scores technique, using G3 as a *S. mansoni* positivity threshold. We suggest that if ≤2% of all POC-CCA+ infections are ++/+++ or ≤0·5% G-Score are G7 and above, it is likely that EPHP has been reached.

## Supplementary Material

The Supplementary Material for this article can be found online at: https://www.frontiersin.org/articles/10.3389/fitd.2022.825721/full#supplementary-material.

Supplementary Material

## Data Availability

Publicly available datasets were analyzed in this study. These data can be found here: https://github.com/iamjessclark/calibration_model.

## References

[1] Chitsulo L, Loverde P, Engels D (2004). Schistosomiasis. Nat Rev Microbiol.

[2] Colley DG, Bustinduy AL, Secor WE, King CH (2014). Human Schistosomiasis. Lancet.

[3] World Health Organization (2020). A Road Map for Neglected Tropical Diseases 2021-2030.

[4] Montresor A, Crompton David WT, Hall A, Bundy DAP, Savioli L (1998). Guidelines for the Evaluation of Soil-Transmitted Helminthiasis and Schistosomiasis at Community Level: A Guide for Managers of Control Programmes/ A Montresor...[, et al.].

[5] Engels D, Sinzinkayo E, De Vlas SJ, Gryseels B (1997). Intraspecimen Fecal Egg Count Variation in *Schistosoma mansoni* Infection. Am J Trop Med Hyg.

[6] Lamberton PHL, Kabatereine NB, Oguttu DW, Fenwick A, Webster JP (2014). Sensitivity and Specificity of Multiple Kato-Katz Thick Smears and a Circulating Cathodic Anttigen Test for *Schistosoma mansoni* Diagnosis Pre- and Post-Reated Praziquantel Treatment. PloS Neglect Trop Dis.

[7] World Health Organization (2020). Enhancing Implementation of Schistosomiasis Control and Elimination Programmes.

[8] Van Lieshout L, Polderman AM, Deelder AM (2000). Immunodiagnosis of Schistosomiasis by Determination of the Circulating Antigens CAA, and CCA in Particualr in Individuals With Recent of Light Infections. Acta Trop.

[9] Casacuberta Partal M, Beenakker M, De Dood CJ, Hoekstra PT, Kroon L, Kornelis D (2021). Specificity of the Point-Of-Care Urine Strip Test for Schistosoma Circulating Cathodic Antigen (POC-CCA) Tested in Non-Endemic Pregnant Women and Young Children. Am J Trop Med Hyg.

[10] Graeff-Teixeira C, Favero V, Pascoal VF, De Souza RP, Rigo FV, Agnese LHD (2021). Low Specificity of Point-of-Care Circulating Cathodic Antigen (POCCCA) Diagnostic Test in a Non-Endemic Area for *Schistosomiasis mansoni* in Brazil. Acta Trop.

[11] Clark J, Moses A, Nankasi A, Faust CL, Moses A, Ajambo D (2021). Reconciling Egg- and Antigen-Based Estimates of *Schistosoma mansoni* Clearance and Reinfection: A Modelling Study. Clin Infect Dis.

[12] Utzinger J, Becker SL, Van Lieshout L, Van Dam GJ, Knopp S (2015). New Diagnostic Tools in Schistosomiasis. Clin Microbiol Infect.

[13] Danso-Appiah A, Minton J, Boamah D, Otchere J, Asmah RH, Rodgers M (2016). Accuracy of Point-of-Care Testing for Circulatory Cathodic Antigen in the Detection of *Schistosome* Infection: Systematic Review and Meta-Analysis. Bull World Health Organ.

[14] Prada JM, Touloupou P, Adriko M, Tukahebwa EM, Lamberton PHL, Hollingsworth TD (2018). Understanding the Relationship Between Egg- and Antigen-Based Diagnostics of *Schistosoma mansoni* Infection Pre- and Post-Treatment in Uganda. ParasitVectors.

[15] Clements MN, Donnelly CA, Fenwick A, Kabatereine NB, Knowles SCL, Meite A (2017). Interpreting Ambiguous 'Trace' Results in *Schistosoma mansoni* CCA Tests: Estimating Sensitivity and Specificity of Ambiguous Results With No Gold Standard. PloS Neglect Trop Dis.

[16] Barenbold O, Garba A, Colley DG, Fleming FM, Haggag AA, Ramzy RMR (2018). Translating Preventive Chemotherapy Prevalence Thresholds for *Schistosoma mansoni* From the Kato-Katz Technique Into the Point-of-Care Circulating Cathodic Antigen Diagnostic Test. PloS Neglect Trop Dis.

[17] Clements MN, Corstjens P, Binder S, Campbell CH, De Dood CJ, Fenwick A (2018). Latent Class Analysis to Evaluate Performance of Point-of-Care CCA for Low-Intensity *Schistosoma mansoni* Infections in Burundi. Parasit Vectors.

[18] Casacuberta-Partal M, Hoekstra PT, Kornelis D, Van Lieshout L, Van Dam GJ (2019). An Innovative and User-Friendly Scoring System for Standardised Quantitative Interpretation of the Urine-Based Point-of-Care Strip Test (POC-CCA) for the Diagnosis of Intestinal Schistosomiasis: A Proof-of-Concept Study. Acta Trop.

[19] Standley CJ, Lwambo NJ, Lange CN, Kariuki HC, Adriko M, Stothard JR (2010). Performance of Circulating Cathodic Antigen (CCA) Urine-Dipsticks for Rapid Detection of Intestinal Schistosomiasis in Schoolchildren From Shoreline Communities of Lake Victoria. Parasit Vectors.

[20] Castro N, Medina R, Sotelo J, Jung H (2000). Bioavailability of Praziquantel Increases With Concomitant Administration of Food. Antimicrob Agents Chemother.

[21] Barenbold O, Raso G, Coulibaly JT, N'goran EK, Utzinger J, Vounatsou P (2017). Estimating Sensitivity of the Kato-Katz Technique for the Diagnosis of *Schistosoma mansoni* and Hookworm in Relation to Infection Intensity. PloS Neglect Trop Dis.

[22] Lindholz CG, Favero V, Verissimo CM, Candido RRF, De Souza RP, Dos Santos RR (2018). Study of Diagnostic Accuracy of Helmintex, Kato-Katz, and POC-CCA Methods for Diagnosing Intestinal Schistosomiasis in Candeal, a Low Intensity Transmission Area in Northeastern Brazil. PloS Neglect Trop Dis.

[23] R Core Team (2018). R Foundation for Statistical Computing.

[24] Denwood MJ (2016). Runjags: An R Package Providing Interface Utilities, Model Templates, Parallel Computing Methods and Additional Distributions for MCMC Models inJAGS. J Stat Software.

[25] Sing T, Sander O, Beerenwinkel N, Lengauer T (2005). ROCR: Visualizing Classifier Performance in R. Bioinformatics.

[26] Lamberton PH, Faust CL, Webster JP (2017). Praziquantel Decreases Fecundity in Schistosoma mansoni Adult Worms That Survive Treatment: Evidence From a Laboratory Life-History Trade-Offs Selection Study. Infect Dis Poverty.

[27] Mccusker P, Rohr CM, Chan JD (2021). *Schistosoma mansoni* Alter Transcription of Immunomodulatory Gene Products Following *In Vivo* Praziquantel Exposure. PloS Negl Trop Dis.

[28] Wiegand RE, Secor WE, Fleming FM, French MD, King CH, Montgomery SP (2021). Control and Elimination of Schistosomiasis as a Public Health Problem Thresholds Fail to Differentiate Schistosomiasis Morbidity Prevalence in Children. Open Forum Infect Dis.

[29] Mawa PA, Kincaid-Smith J, Tukahebwa EM, Webster JP, Wilson S (2021). Schistosomiasis Morbidity Hotspots: Roles of the Human Host, the Parasite and Their Interface in the Development of Severe Morbidity. Front Immunol.

[30] Ayabina D, Kura K, Toor J, Graham M, Anderson RM, Hollingsworth TD (2021). Maintaining Low Prevalence of *Schistosoma mansoni*: Modelling the Effect of Less Frequent Treatment. Clin Infect Dis.

[31] Toor J, Truscott JE, Werkman M, Turner HC, Phillips AE, King CH (2019). Determining Post-Treatment Surveillance Criteria for Predicting the Elimination of *Schistosoma mansoni* Transmission. Parasit Vectors.

