# Supplementary material for: Translating From Egg- to Antigen-Based Indicators for Schistosoma mansoni Elimination Targets: A Bayesian Latent Class Analysis Study
Source: Front Trop Dis. Author manuscript; Available in PMC 2022 Jul 2. (PMC7612949; doi:10.3389/fitd.2022.825721)
Supplement: Supplementary Material [file EMS146323-supplement-Supplementary_Material.pdf]

Supplementary Material

Translating from egg- to antigen-based indicators for *Schistosoma mansoni* elimination targets: A Bayesian latent class analysis study.

Data 2
Model details 2
Infection status 2
Infection intensity 4
Data likelihoods 4
Sensitivity Analysis 7
Simulation details 9
References 11

Jessica Clark[1], Arinaitwe Moses [2], Andrina Nankasi[2], Christina L. Faust[1], Moses Adriko[2], Diana Ajambo[2†], Fred Besigye[2], Aaron Atuhaire[2], Aidah Wamboko[2], Candia Rowell[2], Lauren V. Carruthers[1], Rachel Francoeur[1,3], Edridah M. Tukahebwa[2], Poppy H. L. Lamberton[1*] & Joaquin M. Prada[4*]

[†] Deceased

*Equal contribution as senior authors.

1. Wellcome Centre for Integrative Parasitology, Institute of Biodiversity, Animal Health & Comparative Medicine, University of Glasgow, Glasgow, UK
2. Vector Control Division, Ministry of Health, Kampala, Uganda
3. Faculty of Medicine & Life Sciences, University of Chester, Chester, UK
4. Faculty of Health & Medical Sciences, University of Surrey, Guildford, UK

Here, we present methodological details to accompany those presented in the main text of *Translating from egg- to antigen-based indicators for Schistosoma mansoni elimination targets: A Bayesian latent class analysis study.*

Data

In March 2017, 30 school-aged children aged between six and 14 (270 total) with an even sex distribution were recruited at Bugoto Lake View Primary School, Mayuge District, Uganda. In this study we focus on 210 of these children that provided sufficient stool samples for Kato-Katz and urine for POC-CCAs pre-treatment with praziquantel and albendazole, three-weeks, nine-weeks and six-months post-treatment. Kato-Katz were independently read by highly trained personnel, POC-CCAs had two readers per test, one of which was consistent throughout all tests. Of these children, 55 were also part of a separate cohort study conducted in parallel, where their infection status was parasitologically investigated twice a week for 6-months. Whilst we have Kato-Katz data at the nine-week post-treatment timepoint for the full cohort including the 55, it is only for these 55 children that we also have nine-week POC-CCA data. In short, the sample size for the POC-CCA at nine-weeks post-treatment was 55. Not all children 210 were present pre-treatment, three-weeks and six-months post-treatment either. However, these missing data do not pose a problem to this analysis because the Bayesian framework probabilistically infers missing data. This missing data, however, does not contribute to the likelihood (model fitting process). Removal of children with incomplete data does not improve the fit of the model, but rather increases uncertainty due to reduced information.

Model details

We adapted [1] a discrete time latent class model, which was fit using the R (v. 4.0.2) package, *Runjags* [2,3]. The mathematical details are as follows.

Infection status

The model estimates the unobservable infection status for individual at each timepoint (pre-treatment, three-weeks, nine-weeks and six-months post-treatment). The status of individual i at time t is drawn from a Bernoulli distribution with the probability P_t estimated by the model.

$$Status_{i,t} = Bernoulli(P_t)$$

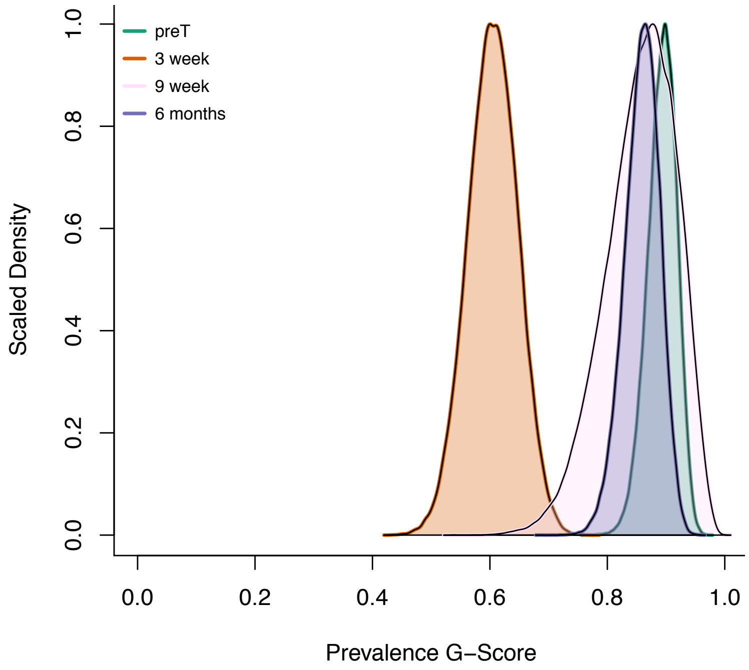

Figure S 1. Posterior distributions for the prevalence estimates per timepoint using the G-Score model

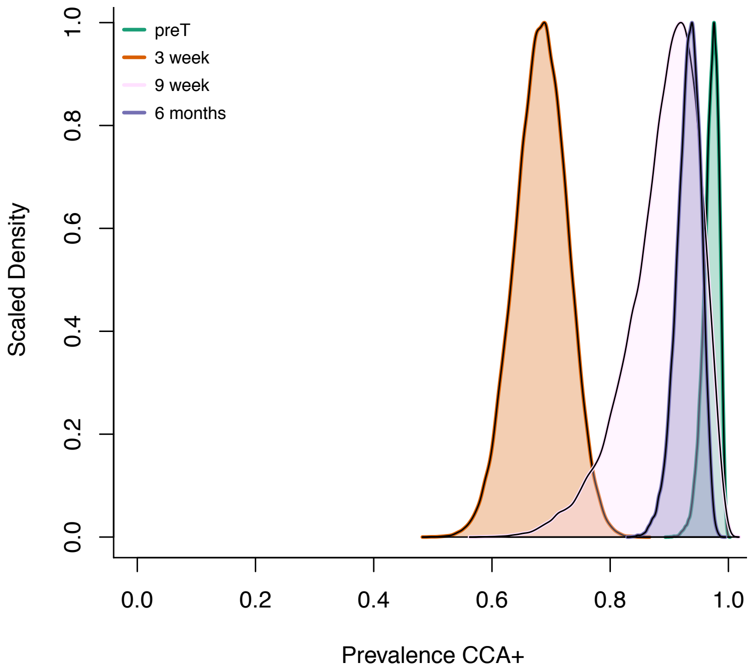

Figure S 2. Posterior distributions for the prevalence estimates per timepoint using the POC-CCA+ model

The status of individual i at time t is denoted as

$$Status_{i,t} = \begin{cases} 0 & uninfected\ or\ undetectable\ infection \\ 1 & infected \end{cases}$$

Infection intensity

We assume that the true infection intensity for individual i at time t ($\lambda_{i,t}$) for those who are negative has to be 0 (top value), whilst the infection intensity when infected is drawn from a Gamma distribution with parameters α and β estimated by the model.

$$\lambda_{i,t} = \begin{cases} 0 \\ Gamma(\alpha_t, \beta_t) \end{cases}$$

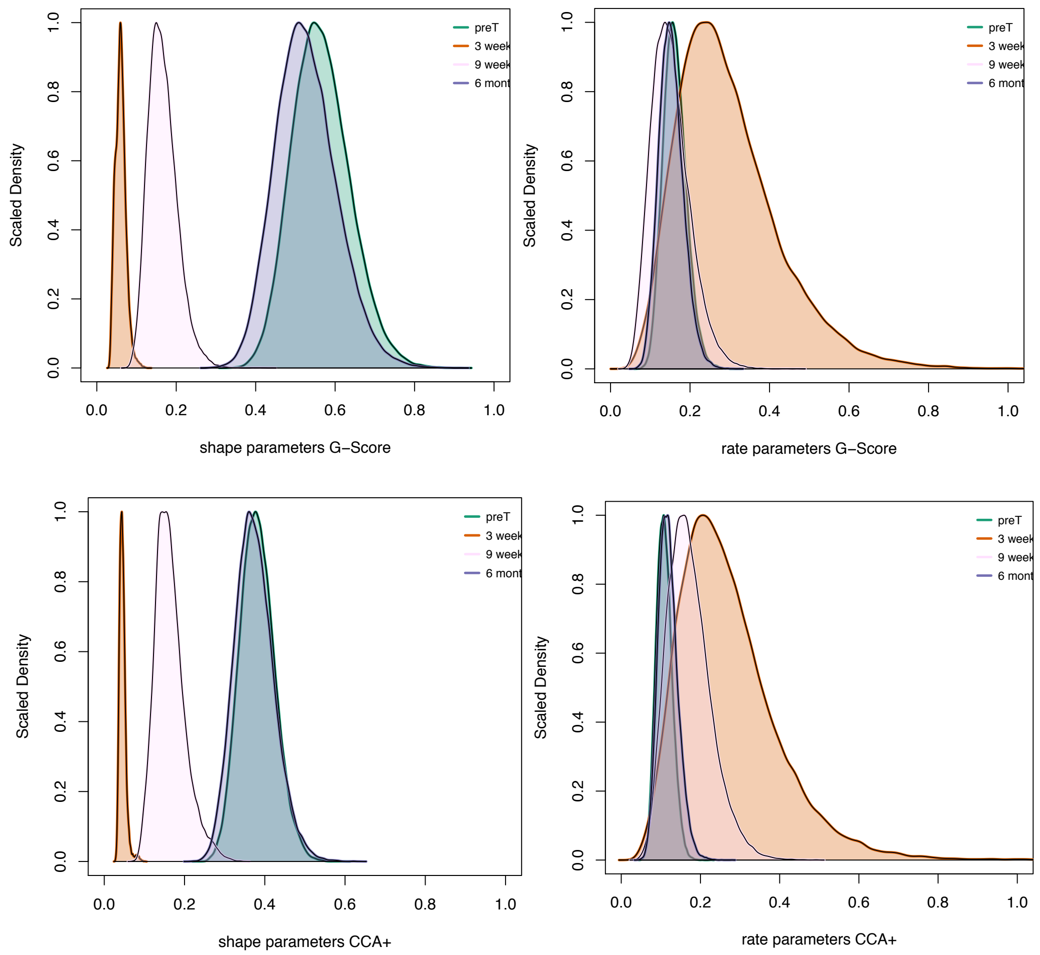

Figure S 3. Posterior distributions for the shape (alpha) and rate (beta) parameters estimated by the G-Score model (top row) and POC-CCA+ model (bottom row).

Data likelihoods

For the Kato-Katz data, each individual i presented a repeat set of stool samples, that produced a maximum of six repeated slides, r at time t giving

$$Kato - Katz_{i,t,r} = \begin{cases} 0 \\ NB\left(\dfrac{\alpha_2}{\lambda_{i,t} + \alpha_2}, \alpha_2\right) \end{cases}$$

Again, we assume that someone who is not infected (top value) must have zero eggs. We assume that the variation seen between and within samples is generated by a Gamma-Negative Binomial process. The shape parameter α_2 was estimated by the model.

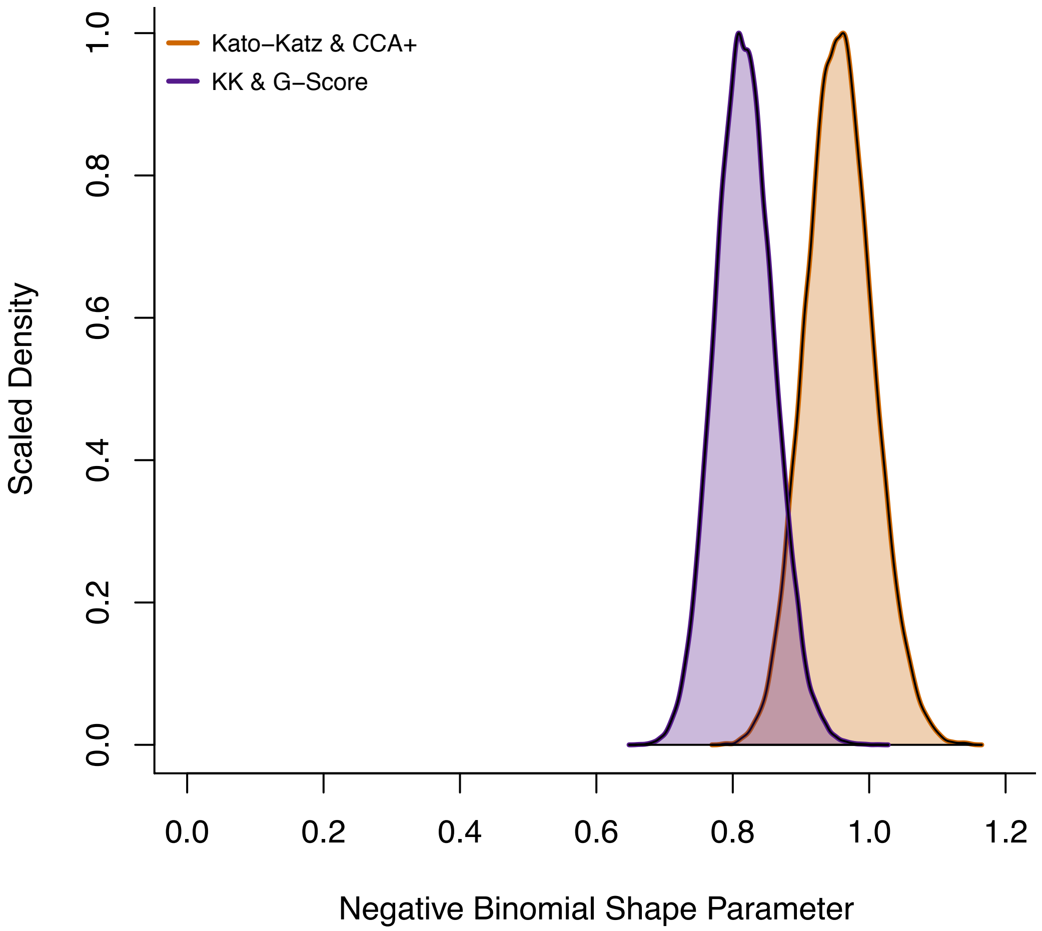

Figure S 4. Posterior distributions of the shape parameters estimated by the POC-CCA+ (orange) and G-Score (purple) models

The POC-CCA data were modelled to reflect the assumption that the higher the infection intensity (i.e., $\lambda_{i,t}$) the higher the POC-CCA score. We do not make the assumption that the relationship is linear but use a logistic function that can take a linear form is this reflects the functional shape of the relationship between the unobservable infection intensity and the POC-CCA scores. This approach is flexible and makes minimal assumptions regarding the interpretation of the POC-CCA+ Trace score or the G2 and G3 scores. The numerator of the logistic function is the maximum value that the POC-CCA diagnostic can return. We transformed the discrete scores into numeric semi-quantitative

integers; 0-4 to represent Negative, Trace, +, ++ and +++ of the POC-CCA+ method, and 0-9 for the G1 (negative)-G10 (highest positive score).

For the POC-CCA+ the likelihood took the following form

$$CCA +_{i,t} = \begin{cases} \mathcal{N}(0, 3.47182) \\ \mathcal{N}\left(\frac{4}{1 + (\lambda_{i,t} - x_0)^{-k}}, 3.47182\right) \end{cases}$$

Whilst the G-Score likelihood was given by

$$G - Score_{i,t} = \begin{cases} \mathcal{N}(0, 1.245443) \\ \mathcal{N}\left(\frac{9}{1 + (\lambda_{i,t} - x_0)^{-k}}, 1.245443\right) \end{cases}$$

The logistic growth rate $-k$ and the sigmoidal intercept x_0 were estimated by the model.

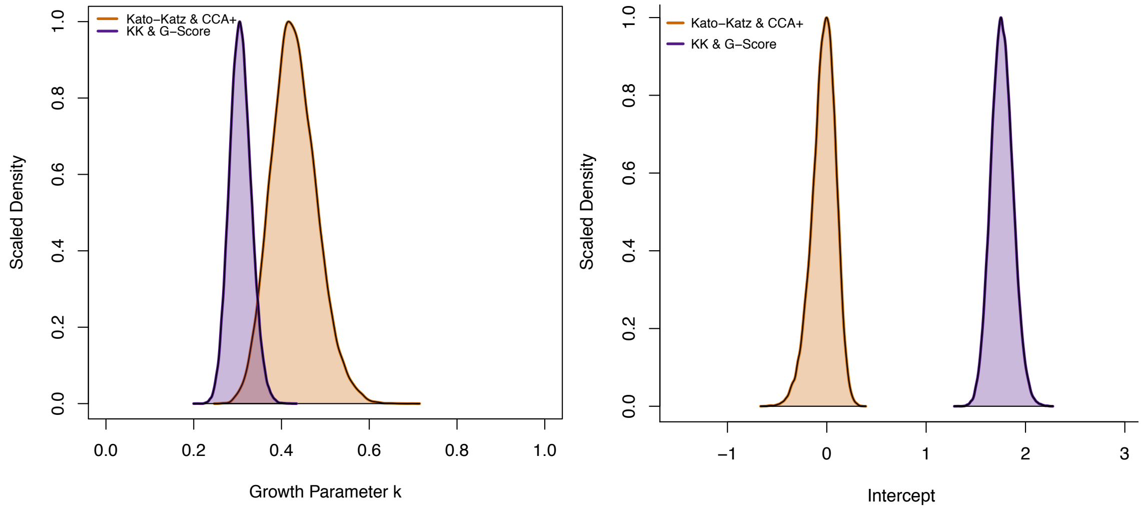

Figure S 5. Posterior distributions for the growth (k) and intercept parameters estimated by the POC-CCA+ model (orange) and the G-Score model (purple).

The scores were drawn from a normal distribution to further add noise to account for reader variation. This allows for those who are not infected to still be given a score of Trace or G2/G3. The intercept x_0 and growth k parameters were estimated by the model.

Sensitivity Analysis

Ideally they would be estimated independently at each timepoint as it is conceivable that the relationship between egg and antigen changes with treatment (as indicated by our results, see figure 4 , main text). We lacked the data to do this, however. Additionally, the precision variables here are fixed values calculated from the data, reflecting the distribution of POC-CCA scores across the range of infection intensities. Allowing the model to estimate this parameter for the POC-CCA+ data did not result in a satisfactory fit as indicated in figure S6, for example by the bimodal posterior distribution at three weeks. Attempting to estimate this parameter with the G-Score model framework was not possible.

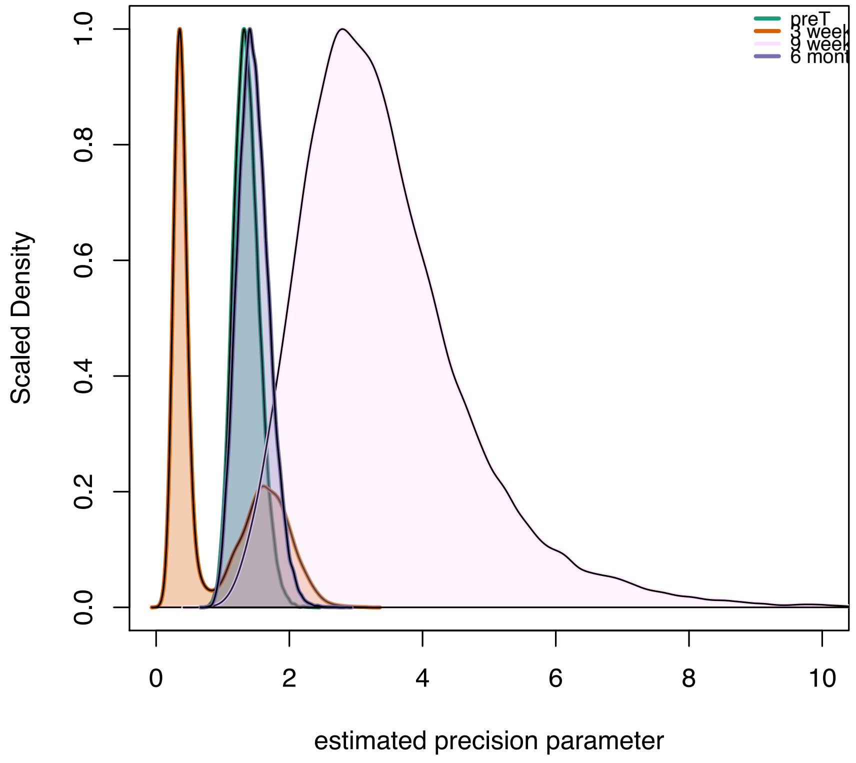

Figure S 6. Posterior distributions per time point for the estimated precision on the POC-CCA scores drawn from a normal distribution. The three week posterior distribution is bimodal indicating a poor fit.

We also reduced the precision (i.e., increased the variance) on the normal draw for the POC-CCA scores, such that instead of allowing one standard deviation from the mean from the distribution of scores across infection intensity, we allowed for two standard deviations from the mean. The precision parameter then took on the value of 0.87 for POC-CCA+ and 0.31 for G-Score. This value resulted in an unsuitable likelihood on the G-Score data, such that the model did not run. The POC-

CCA+ did run, however, using the estimated probability of infection as an example (figure S7) it is evident that the estimates are diffuse and thus the model fit not as precise.

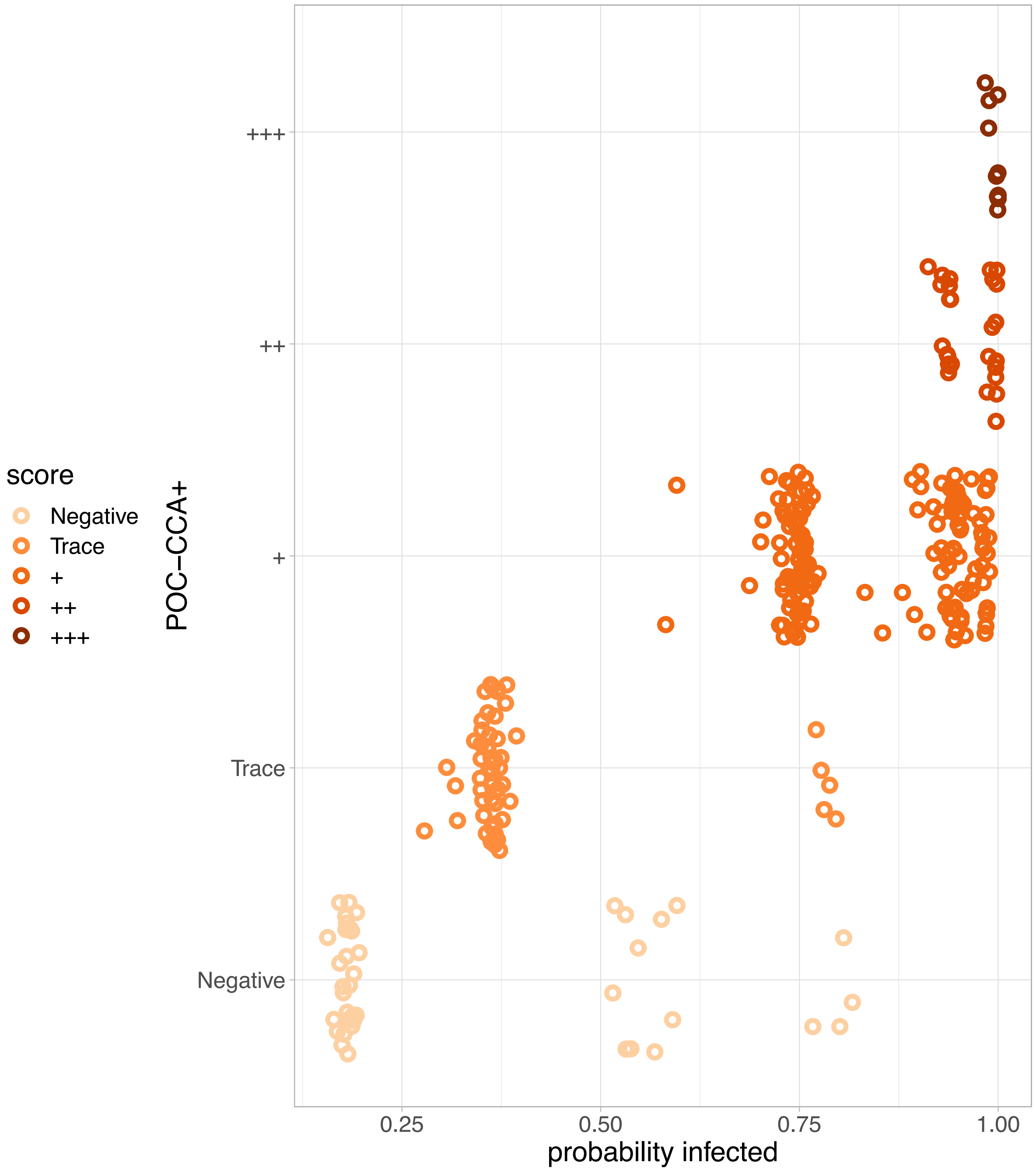

Figure S 7. Predicted probabilities of infection associated with each score when the fixed precision parameter is calculated to allow POC-CCA scores that are two standard deviations from the mean. The fit is poor as the estimates are diffuse.

Simulation details

Infection status was drawn from a binomial distribution (0/1) with probability = the proportional prevalence. For individuals with Status = 0 (uninfected), true egg counts must be 0, assuming 100% specificity. For those with Status =1 (infected) infection intensity was simulated so that 1% of all infections were ≥400epg as per the WHO current EPHP threshold target. The simulation emulated the model, with infection intensity (λ) drawn from a gamma distribution, where the shape and rate parameters of the gamma distribution were estimated using R's *optim* function, to provide the EPHP distribution of infection intensities. The rate parameter was estimated, and the shape parameter calculated as $\frac{mean}{rate}$ with the mean value taken from literature.[4] Two Kato-Katz counts from a single sample were simulated, with each individual's egg count drawn from a negative binomial distribution, with the gamma distributed mean and the over-dispersion parameter randomly sampled from the G-Score and POC-CCA+ models described above, for each respective simulation. As in the models above, those with Status = 0 were allowed Trace scores or G2/G3 (i.e., false positives[5]). For those with Status =1, the scores were drawn from a truncated normal distribution with a logistic function as the mean, where the *k* and *intercept* parameters were joint-randomly sampled from the model posteriors.

Our simulations show that in low prevalence settings that have achieved EPHP, using + as the POC-CCA+ threshold slightly underestimated prevalence (figure S8 , orange) in keeping with the ROC curves. For comparison, and to illustrate the impact of considering Trace as positive according to our results, we show the inflated estimated prevalence when using Trace as positive (figure S8 , blue). A G3 score consistently over estimated prevalence by about 7.5% (figure S8 , purple).

Figure S 8. Predicted prevalence correlated to true prevalence based on simulations. G-score (purple) uses G3 as the first positive score. POC-CCA+ (orange) uses + as the first positive score. POC-CCA+ with Trace as positive is in blue. Kato-Katz is in grey. The dashed line indicates the diagonal.

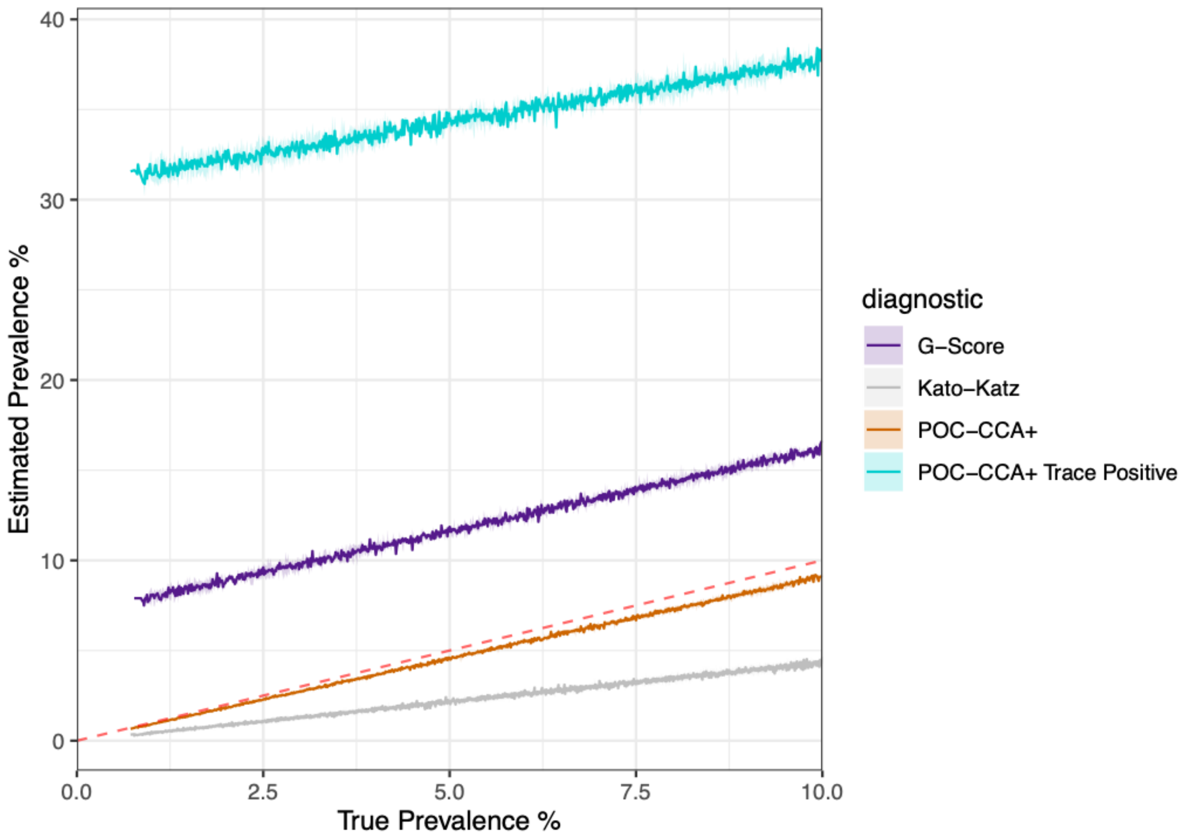
Estimated Prevalence %
40
30
20
10
0
0.0
2.5
5.0
7.5
10.0
True Prevalence %
diagnostic
G–Score
Kato–Katz
POC–CCA+
POC–CCA+ Trace Positive

References

1. Clark J, Arinaitwe M, Nankasi A, et al. Reconciling egg- and antigen-based estimates of Schistosoma mansoni clearance and reinfection: a modelling study. Clinical Infectious Diseases 2021.
2. Denwood MJ. runjags: An R Package Providing Interface Utilities, Model Templates, Parallel Computing Methods and Additional Distributions for MCMC Models inJAGS. Journal of Statistical Software 2016;71.
3. R Core Team. R: A language and environment for statistical computing. R Foundation for Statistical Computing. Vienna, Austria 2018.
4. Ruberanziza E, Wittmann U, Mbituyumuremyi A, et al. Nationwide Remapping of Schistosoma mansoni Infection in Rwanda Using Circulating Cathodic Antigen Rapid Test: Taking Steps toward Elimination. American Journal of Tropical Medicine and Hygiene 2020;103:315-24.
5. Casacuberta Partal M, Beenakker M, de Dood CJ, et al. Specificity of the Point-of-Care Urine Strip Test for Schistosoma Circulating Cathodic Antigen (POC-CCA) Tested in Non-Endemic Pregnant Women and Young Children. The American Journal of Tropical Medicine and Hygiene 2021;104:1412-7.
